# Advances and Computational Tools towards Predictable Design in Biological Engineering

**DOI:** 10.1155/2014/369681

**Published:** 2014-08-03

**Authors:** Lorenzo Pasotti, Susanna Zucca

**Affiliations:** ^1^Laboratory of Bioinformatics, Mathematical Modelling and Synthetic Biology, Department of Electrical, Computer and Biomedical Engineering, University of Pavia, 27100 Pavia, Italy; ^2^Centre for Tissue Engineering, University of Pavia, 27100 Pavia, Italy

## Abstract

The design process of complex systems in all the fields of engineering requires a set of quantitatively characterized components and a method to predict the output of systems composed by such elements. This strategy relies on the modularity of the used components or the prediction of their context-dependent behaviour, when parts functioning depends on the specific context. Mathematical models usually support the whole process by guiding the selection of parts and by predicting the output of interconnected systems. Such bottom-up design process cannot be trivially adopted for biological systems engineering, since parts function is hard to predict when components are reused in different contexts. This issue and the intrinsic complexity of living systems limit the capability of synthetic biologists to predict the quantitative behaviour of biological systems. The high potential of synthetic biology strongly depends on the capability of mastering this issue. This review discusses the predictability issues of basic biological parts (promoters, ribosome binding sites, coding sequences, transcriptional terminators, and plasmids) when used to engineer simple and complex gene expression systems in* Escherichia coli*. A comparison between bottom-up and trial-and-error approaches is performed for all the discussed elements and mathematical models supporting the prediction of parts behaviour are illustrated.

## 1. Background

In order to handle complexity in the design of customized systems, engineers usually rely on a bottom-up approach: components are quantitatively characterized and the output of an interconnected system is predicted from the knowledge of individual parts function [1]. This process is applied in all the fields of engineering and is useful to hide the complexity of the individual components functioning, thus using them as input-output modules [2].

This strategy is successful only in a modular framework, where parts behaviour does not change upon interconnections and, in general, when the same parts are reused in a different context [3, 4]. Even if this property does not persist, the bottom-up approach is still feasible when engineers are able to predict how parts behaviour varies as a function of environmental changes or interconnections [5]. In electronics, examples of the latter situation are resistors: they are characterized by an electrical resistance, which does not change upon connection in different circuits. However, it is well established that resistance changes as a function of temperature and, for this reason, datasheets of electric components report the temperature-resistance characteristic in order to make the output of complex circuits predictable when used in different environments. Another example is a circuit with a nonzero impedance; it can exhibit a different input-output behaviour when interconnected to different loads. However, it is still possible to predict the output of such interconnected systems since mathematical models of electrical circuits are able to describe voltage and current throughout the network.

Mathematical models are widely used in many areas of engineering to support the early design steps of a system, guide the debugging process, measure nonobservable parameters, and finally predict the quantitative behaviour of systems composed by precharacterized parts. Likewise, models also play an important role in a biological systems framework; in fact, they are often used to study complex metabolic interactions, like those occurring in disease conditions to understand the underlying processes and/or predict the effect of drugs [25]. Some mathematical models of biological/physiological systems have also been approved by the US Food and Drug Administration (FDA) for use in simulated clinical trials, thus enabling researchers, for example, to support or even skip expensive* in vivo* trials [26].

Synthetic biology aims to realize novel complex biological functions with the same principles on which engineering disciplines lay their foundations: modularity, abstraction, and predictability [2, 27, 28]. As a result, synthetic biologists so far have mainly focused on the definition of biological parts and on their abstraction and standardization, in order to deal with well-defined components with specific function [29]. This process has brought to the creation of biological parts repositories including DNA parts that can be shared by the scientific community, like the MIT Registry of Standard Biological Parts [30–32], to standardized and easy-to-automate DNA assembly strategies [33–35], and to standard measurement methodologies to share characterization results of parts, like promoters [36, 37]. Researchers have also focused on the realization of engineering-inspired functions to learn the complexity that could be reached in a biological context. Towards this goal, researchers built up devices that implement logic gates and functions [19, 38–41], memories [42], oscillators [43–45], other waveform generators [46, 47], signal processing devices [48–50], and the like. Many of them relied on mathematical models to support the early design steps and to capture the behaviour of the designed circuit. For example, two of the synthetic biology milestones are a genetic toggle switch [42] and an oscillator (the* repressilator*) [43], both implemented in* Escherichia coli* via genetic networks of properly connected transcriptional regulators. A semiquantitative investigation of the features required for a correct circuit behaviour was performed via mathematical models, by using dimensionless equations or reasonable parameter values. Thanks to the model analysis, the authors could learn useful guidelines for correct design of circuits exhibiting the desired functioning, for example, fast degradation rates of repressor proteins encoded in the oscillatory network [43].

The realization of complex functions has brought to some biological systems of high impact. An engineered pathway was implemented in recombinant yeast to produce the antimalarial drug precursor artemisinin [51]; a biosensor-encoding genetic device was implemented in microbes to detect arsenic in drinking water and to provide a colour change of its growth medium as visual output [52, 53]; microbes were recently engineered to produce bioethanol from algal biomass [54] or advanced fuels from different substrates [55].

However, despite many examples of complex engineering-inspired function implementation and also of industrially relevant solutions to global health, environmental, and energy problems, a rigorous bottom-up design process is not currently adopted because the predictability boundaries still have to be clearly defined [3, 56, 57]. The high potential of synthetic biology strongly depends on the achievement of such task [58]. Trial-and-error approaches represent an alternative: if synthetic biologists cannot design a system from the bottom-up, they can rely on random approaches, where, for example, circuit components are mutated and the best candidate implementing the function of interest is selected [38, 59, 60]. Depending on the reliability of predictions and of mathematical models, this process could be completely random or partially guided. In general, trial-and-error approaches are time- and resource-consuming, and are characterized by a low efficiency. However, recent advances in the construction of biological systems, for example, DNA and/or strain production via automated procedures, may provide a good alternative to the rational bottom-up approach, especially when accurate, automated, and possibly low-cost screening methods are available to rapidly evaluate the output of the constructed circuits [60].

This review discusses the predictability issues of basic biological parts (promoters, ribosome binding sites—RBSs, coding sequences, transcriptional terminators, and plasmids) when used to design the desired biological function in the form of a simple or complex gene expression system. Even though synthetic biological systems may be implemented in several organisms (or even* in vitro *[61]) and may have disparate architectures and regulatory mechanisms [62, 63], the review will focus on predictability of parts* in vivo* in the* E. coli* bacterium, according to the biological information flow described in the central dogma of molecular biology [64]: protein-coding DNA sequences (herein called genes) are transcribed into mRNA molecules, which are converted into proteins by ribosomes, and, finally, DNA sequences can be replicated in living cells to propagate the encoded function to the progeny. Thus, in the considered framework, the possible basic architectures are shown in Box 1: promoters can trigger the expression of a single gene (monocistronic architecture) or a set of genes (polycistronic or operon architecture), each gene is transcribed with its RBS upstream and finally terminators stop transcription. Ribosomes complete the process by translation of mRNA molecules into the proteins of interest, from the start codon (generally AUG) to the stop codon (generally UAA). Complex genetic circuits can be realized with a set of such gene expression units, implementing the interactions of interest and giving the desired product as output. Genetic circuits can be placed on a plasmid vector or otherwise they can be integrated into a target position of the bacterial chromosome.

Even if other classes of parts can be used to construct complex genetic systems and other elements can also affect circuit behaviour, we will focus only on the abovementioned genetic parts and architectures, given a specific strain and environment. Other important contexts, like the host (the reciprocal variation of parts behaviour and host metabolism when a circuit is incorporated), environmental (the reciprocal variation of parts behaviour and environmental parameters), ecological (changes of synthetic circuit and surrounding community parameters, as well as strains fitness), and evolutionary (changes of DNA composition) contexts are reviewed elsewhere [57]. Other reviews are complementary to the present work, describing software tools for parts/pathway identification [65] and cellular behaviour modelling at different scales [65–67].

Each of the biological parts and architectures described in Box 1 will be considered. We will discuss to which extent their function can be predictable and then a comparison between bottom-up and trial-and-error approaches will be carried out. For each part and architecture, the contribution of mathematical models supporting the prediction of circuit behaviour will be highlighted. Even though many computer-aided design (CAD) tools are available for synthetic circuits [68], only mathematical analysis tools (also including tools from the field of systems biology) and predictive models of parts function will be considered, while no software tool for database access/development or for the assembly process support [65] will be taken into account. In particular, the considered tools can be ordinary differential equation (ODE) models (or derived steady-state equation models) based on empirical or mechanistic functions, or predictive models able to infer parts behaviour given their sequence and/or their DNA context.

## 2. Research Studies and Tools to Support Bottom-Up Design

The kit of parts, architectures, and contexts available to synthetic biologists will be discussed. Then, interconnection issues will be considered. A summary of the selected methods and tools available for parts/devices quantitative prediction is reported in Table 1.

### 2.1. Promoters

Promoters are intrinsically context-dependent parts, since it is known that their upstream and downstream elements may affect transcriptional activity [69–73]. The research studies on the predictability of promoters have focused on their context-dependent variability and on activity prediction given their nucleotide sequence. Context-dependent variability studies aim to evaluate whether promoters show the same activity in different contexts, for example, when promoters have different sequences upstream, when expressing different genes/mRNAs, or when other independent gene expression cassettes are present in the same circuit. Generally, the activity of a set of promoters can be indirectly measured via reporter proteins, provided that the downstream sequences are the same (i.e., identical RBSs, reporter genes, terminators, and similar transcription start sites—TSSs) so that mRNA primary and secondary structures do not significantly vary among the promoter measurement systems [37]. Using the same architecture, the activity can be evaluated via qPCR, by directly measuring the mRNA level [74]. Davis et al. [73] quantified a set of constitutive promoters and found that activity was affected up to 4-fold when a specific upstream (UP) sequence is placed before promoters, even though in some cases activity was not affected. Other studies showed that the upstream sequence-dependent activity change could be as high as 300-fold and the consensus sequences that can affect such different transcriptional activity were identified [72, 75, 76]; this effect was observed when using the rrnB P1 promoter, but activity change was also observed for the lac promoter. On the other hand, specific “anti” sequences downstream of promoters can limit the RNA polymerase escape process, thus affecting promoter activity [77]; such elements were found to decrease sigma70 and sigma32 promoter activity up to 10-fold [69]. Davis et al. also tested the effects of different sequences flanking promoters downstream, including an “anti” sequence or different reporter genes (GFP, dsRed, and Gemini) with the same RBS, yielding an activity change up to 2-fold [73]. A similar fold change was observed in analogous experiments, where Martin et al. [78] tested GFP, lacZ-alpha, and Gemini as reporter genes. In their work, however, the 2-fold difference persists for the strongest promoter, which might be affected by an excess of the lacZ-alpha fragment compared to the omega fragment needed for complementation. A study of our group [20] yielded a lower estimate of activity change for a set of 5 widely used promoters expressing the green fluorescent protein (GFP) with the BBa_B0032 RBS or the red fluorescent protein (RFP) with the BBa_B0032 or BBa_B0034 RBS: only one of the tested promoters showed a significant activity change among the three conditions, with a coefficient of variation (CV) of 22%. The abovementioned studies expressed promoter activities in RPUs, in order to provide comparable measurements among the different reporters used. Recent advances in DNA synthesis, assembly, and high-throughput characterization techniques enabled the quantification of very large libraries of single gene expression cassettes composed by different promoters, RBSs, and target genes, by measuring the fluorescence of reporter gene, as well as mRNA level via qPCR or next generation sequencing. In particular, Kosuri et al. performed the so far largest scale experimental study, where 114 promoters and 111 RBSs were combined upstream of a GFP gene [60]. Promoters were found to trigger consistent RNA levels of the downstream transcript among the different RBS-gene combinations. By using an ANOVA model for data interpretation, it was found that promoter sequence accounted for about 92% of total variability of mRNA level, demonstrating that promoters are the main factors affecting mRNA level, even though they expressed different mRNAs. RBSs accounted for 4% of total variability, which could be due to transcription rate modulation by the sequence downstream of promoter or to other phenomena not involving transcription, such as RBS-dependent mRNA degradation or sequestration (see discussion in Section 2.2.)

The majority of flanking sequence-dependent studies on promoters are relative to downstream sequences, while upstream sequences are less frequently studied. Even though highly stimulatory or inhibitory effects may be obtained via UP or “anti” sequences, promoters were found to change their activity within a reasonably low fold-change when not flanked by such difficult elements.

Although such data gave a significant contribution towards the understanding of promoter reusability, gene expression systems composed by independent expression cassettes are not similarly well studied and could yield unpredictable effects. Hajimorad et al. [79] studied the mRNA levels produced by different gene expression cassettes to test the superposition of the effects in synthetic biological systems at different copy number levels; they found conditions where even three cassettes could provide predictable levels of mRNA, while, in other configurations, cassettes could not be considered as modular systems. Similarly, our group [20] used two cassette-systems expressing GFP and RFP under the control of a set of promoters, detecting fluorescence as output. Cassette position was also studied. Context-dependent variability was higher than for individual cassette expressing different reporters (maximum CV of 33% versus 22%). A part of this variability could be explained by a different upstream sequence; that is, promoters could be flanked by the transcriptional terminator of the upstream cassette or by the plasmid sequence upstream of the cloning site.

Activity prediction studies given the nucleotide sequence of promoters have not yet produced accurate tools for the widely used sigma70 promoters. Promoter strength can be affected by many sequence features, which are not completely understood yet, including the −35/−10 sequences, the spacer between them and the above discussed flanking sequences. Recent efforts towards prediction include the works of Rhodius et al. [6, 80], who developed position weight matrix-based models to predict the activity of sigmaE promoters as a function of their sequence, as well as their flanking sequences (UP elements), with good predictive performance (*r* = 0.86 after cross-validation) [6]. However, the same methods are not likely to work for sigma70 promoters due to their complex structure [80]. De Mey et al. used partial least squares (PLS) regression to classify promoter strength as a function of nucleotide sequence [7]; this approach accurately predicted the activity of 6 out of 7 promoters used as a test set. Meng et al. developed an artificial neural network (ANN) to predict the strength of regulatory elements composed by a promoter and an RBS [8]; this approach brought to the accurate prediction of an initial test set of 10 promoter-RBS pairs (*r* = 0.98) and good performance was also obtained on a second set of 16 newly constructed pairs. The described tools provided promising results but additional work is needed to independently validate such methods on other datasets and to fully understand promoter sequence features.

In summary, reproducible context-dependent variability studies should be performed to fully understand the factors affecting promoter activity in individual expression cassettes and in multiple cassette systems. Large libraries of parts are now affordable and, for this reason, the analysis of such factors will be facilitated, as well as activity prediction given promoter sequence. Standard [37] and multifaceted [74] characterization approaches have been proposed to provide robust measurements that can be shared and reproduced in many laboratories.

### 2.2. RBSs

RBSs are strongly context-dependent elements, since their surrounding sequences can affect ribosome binding and, as a result, the translation initiation rate per transcript. In particular, even a few nucleotide changes in the RBS or in the surrounding sequences can dramatically affect translation [10] and the use of different genes downstream of an RBS can provide completely different translational efficiencies [81]. Given the sequence of a gene and its 5′UTR, biophysical models have been used to predict the translation initiation rate by modelling local and global folding, as well as the interaction between RBS and 16S ribosomal RNA. Computational tools, such as the RBS Designer (stand-alone application, [10]), the RBS Calculator (web-based application, [9]), and the UTR Designer (web-based application, [11]) are available to perform such tasks. They take into account the 5′UTR sequence, as well as the first portion of coding sequence to predict the translation initiation rate level. The RBS Calculator and UTR Designer use similar biophysical thermodynamics-based models, while the RBS Designer uses a steady-state kinetic model of stepwise-occurring reactions [82, 83]. These tools showed similar and reasonably good predictive performance (*r* ^2^ > 0.8) and can also be used to forward-engineer novel RBSs with a desired strength [83]. They differentiate for the use of different external tools for energy computation [83] and for some specific peculiarities; for example, RBS Calculator provides indication of confidence and it is frequently updated [84], RBS Designer considers long-range interactions within RNA and can predict the translation efficiency of mRNAs that may potentially fold into more than one structure, while UTR Designer enables codon editing to minimize secondary structures [83]. Other efforts towards RBS prediction include an artificial neural network, already cited above, to evaluate the strength of promoter-RBS pairs [8].

The RBS Calculator is one of the most commonly used tools in the synthetic biology community: it was used in basic research studies to tune the response of a synthetic AND gate [9], to generate a set of RBSs of graded strengths to evaluate the transcription/translation processes [85], and to test DNA assembly platforms [33, 35], as well as in applied research to optimize biosynthetic pathways [86, 87]. Although it was proved to be useful to guide the choice of proper RBS sequences given a downstream gene, its accuracy is limited and additional tools should be developed to improve the predictability of RBSs [57, 81].

RBSs could also affect the mRNA decay rate by causing different secondary structures [16]. In addition, Kosuri et al. also observed a mutual interaction between transcription and translation: in fact, translation efficiency can affect mRNA levels, probably because the most translated mRNA molecules are protected from degradation, compared to the least translated mRNAs [60].

In summary, as in the case of promoters, large datasets have been useful to show the contributions of different context-dependent factors. Due to the strong context-dependent nature of RBSs, experimental studies mainly focused on flanking sequences, while the evaluation of RBS modularity in complex circuits still needs to be studied.

### 2.3. Genes

Given a target protein, its coding sequence can affect both transcription and translation processes [15, 88]. As described above, mRNA secondary structures could affect mRNA degradation and limit RBS accessibility to ribosomes and, in addition, AT-rich sequences can cause premature transcriptional termination [89]. Codon usage has been reported to affect the translation process [90]. In this framework, most of the efforts towards the prediction of the contribution of gene sequence to transcription/translation processes have focused on the development of gene optimization algorithms. To define them, several sequences need to be constructed to cover a sufficient number of hypotheses; although the cost of synthetic genes is greatly decreasing, gene synthesis still brings to expensive studies [88]. For this reason, the process of sequence optimization is not fully understood and no consensus rules have been found for gene optimization. Some research studies identified strong secondary structures as the primary limiting factors in protein synthesis [91], while other studies did not find a correlation between predicted secondary structure and expression level [92]. On the other hand, in some studies expression level has been found to correlate with the codon adaptation index (CAI) [93, 94], often used to express the codon bias of a gene towards common codons [95], while in other studies this correlation was null [88, 91]. The codon randomization method, where codons are extracted from codon usage frequency tables, was found to be superior to the “one amino acid-one codon” strategy, where the CAI is maximized [15, 92]. Finally, codon context, that is, the influence of codon pair usage, was found to affect protein expression, although no ready-to-use software tool is available to carry out an optimization procedure based on such feature [90].

All the features described above might be gene and variant dependent [88] and, for this reason, several studies should be conducted to identify the correct features of gene sequence affecting transcription, translation, and other processes. In particular, the simultaneous measurement of mRNA and protein level can provide exhaustive data to decouple the effects of gene sequence changes on cellular processes. In a large-scale study, performed by Goodman et al., a library of >14,000 expression systems was constructed to test the contribution of the N-terminal codons on gene expression [96]; they measured DNA, RNA, and protein levels and confirmed that mRNA secondary structure is a crucial factor which can tune gene expression up to ~14-fold.

The research efforts carried out so far have brought to different gene optimization tools, currently used by synthetic biologists and gene synthesis companies to optimize protein expression, according to codon usage frequency tables, global GC content, minimization of hairpin structures within the gene, and/or of secondary structures in the N-terminal codons [97, 98]. The free software tools proposed in literature include, for instance, GeMS (web-based application, [12]), Optimizer (web-based application, [13]), Synthetic Gene Designer (web-based application, [14]), and Gene Designer (stand-alone application, [15]). All the tools mainly differentiate for their available options for designing genes (e.g., avoid unwanted restriction sites and inverted repeats, design framework of oligonucleotides for gene synthesis) and for their codon optimization strategy (e.g., “one amino acid-one codon” method, probabilistic methods, or hybrid solutions, based on codon frequency tables from different sources) to take into account codon usage and constraints. Because many available tools are proprietary of gene synthesis companies, an accurate comparison of the implemented methodologies is not feasible and, in addition, their performances still need to be experimentally evaluated on different gene sets.

In summary, although prediction tools have been proposed, no widely accepted algorithm is available to predict the effects of gene sequence on transcription, translation, or mRNA degradation.

### 2.4. Terminators

Rho-independent terminators are herein considered. Although very efficient terminators are available (e.g., the popular BBa_B0015 double terminator from the MIT Registry of Standard Biological Parts), the repeated use of a small set of elements in a genetic circuit may result in poor evolutionary stability [99, 100]. For this reason, reliable methods to design new terminators with predictable strength and methods to predict the efficiency of already existing terminators given their sequence are required.

Terminator efficiency can be characterized via an operon-structured measurement system, where a promoter drives the expression of two different reporter genes with the terminator sequence to be measured that is assembled between these two genes. The two reporter proteins are quantified and termination efficiency is computed from their values, considering the operon without the terminator of interest as a control [16, 17, 101].

Like promoters and RBSs, also terminator efficiency has been found to be dependent on the surrounding context. In particular, Cambray et al. [16] tested different minimal terminators, including only the hairpin and U-tail sequences and compared their termination efficiency to the respective full-length terminators. Efficiencies significantly changed between the two contexts for almost all the 11 tested terminators, demonstrating that sequences flanking the essential terminator parts are crucial. The authors also used a multiple linear regression model to build up a predictive tool for transcriptional termination given the terminator sequence, using a set of features identified via stepwise regression, but the resulting predictor gave poor performance on the 54 terminators used (*r* = 0.61 after cross-validation). Only by excluding the low efficiency terminators, low predicted folding frequency terminators, and extended terminators classes, the Pearson correlation coefficient *r* increased to 0.85 after cross-validation. Through a complementary approach, Chen et al. [17] experimentally characterized a large set of terminators (582) and analyzed how sequence features contribute to their strength. The dominant features were used to build up a biophysical model that aimed to capture termination strength (Ts) as a function of the U-tract, hairpin loop, stem base, and A-tract-free energies. The model was used to fit via linear regression the experimentally determined Ts, yielding a squared *r* value of 0.4, which results in low predictive performances. Although not currently available to users, the tools developed in the above publications [16, 17] can be implemented through the provided regression coefficients, web-based nucleic acid folding tools, and specific indexes computed from terminator sequences. These two recent studies relied on experimental measurements performed via the abovementioned operon structure with reporter genes. However, Cambray et al. constructed measurement plasmids with RNAse sites flanking the terminator to be measured, in order to avoid terminator-dependent mRNA folding, which might affect the translation efficiencies of the two reporter genes. The authors tested RFP-GFP and GFP-RFP operons with terminators flanked by RNAse III, RNAse E, or nonfunctional RNAse III sites. The configuration giving the lower coefficient of variance for the upstream gene level was the RFP-GFP operon with RNAse III sites, which was used for all the characterization experiments of their paper. Conversely, Chen et al. used a GFP-RFP operon without RNAse sites, since they found that, in their configuration, RNAse E sites presence affected the downstream gene expression. In light of these findings, a standard measurement method for terminators still needs to be defined in order to enable reliable quantifications and to avoid potential mechanisms that may complicate the measurement of terminator efficiency, for example, promoters that might arise at the interface of the terminator to be measured and the downstream gene of the operon [17].

In summary, sequence features affecting terminators behaviour have been recently evaluated on large datasets, but predictive models with good performances are not available yet, demonstrating that different models and additional knowledge on transcriptional termination are needed, as well as a standardized setup for experimental measurements.

### 2.5. Interconnected Networks and Retroactivity

In the philosophy of bottom-up composition of biological systems, arbitrarily complex networks are considered as black-box modules that can be interconnected. Their characterization can provide the essential elements to describe their steady-state and dynamic behaviour. In a modular framework, such knowledge enables the prediction of composite networks functioning. To quantitatively test the modularity boundaries of biological systems, recent studies have focused on the characterization of systems subparts and on the prediction of the behaviour of composite systems, obtained upon their interconnection. Wang et al. [18] tested different regulated promoters (inducible by arabinose, AHL, and IPTG) as the inputs of AND/NAND gates, whose output was visualized via GFP at two different temperatures. After a fitting process involving one specific configuration (i.e., one of the cited input modules), the fluorescence output of the other configurations was predicted from the individual characterization of input devices and AND/NAND gates. Experimental data and predictions exhibited a Pearson correlation coefficient of 0.86 to 0.98, even though some specific input combinations yielded highly different values. Moon et al. [19] constructed and characterized a set of AND gates. Then, they used them to engineer composite two layered logic functions: a 3-input system including 3 input devices connected to two AND gates and a 4-input system including 4 input devices and 3 AND gates. The latter represented one of the largest genetic programs built up so far, with a total of 11 regulatory proteins, 21 kbp-length on three plasmids. The basic AND gates were individually characterized as before and the output of the complex 3- and 4-input systems was predicted and compared with experimental data. The 3-input system yielded a lower deviation between prediction and data, compared to the 4-input system. Our group also faced prediction problems with simple interconnected networks composed by an input device (inducible promoters or constitutive promoters of different strengths) assembled with a TetR-based NOT gate which provides GFP as output [20]. The individual input devices were characterized via RFP measurements and the steady-state transfer function output of the NOT gate driven by each of the input systems was quantified. These data were fitted with a Hill function: they had similar maximum activity and Hill coefficients, while the switch point varied about 44%, which was considered as an estimate of interconnection error with these elements.

The mentioned studies evaluated interconnection-dependent variability in considerably complex systems but they did not characterize the causes of such deviations. One of the best characterized and formalized interconnection errors is retroactivity, a phenomenon that extends the electronic engineering notion of impedance or loading to biological systems [5]. The functioning of a given system can change when a downstream or upstream system is connected, for example, because of unwanted sequestration of transcription factors by the connected modules. In this case, the individual systems cannot be considered to be modular; however, given the knowledge of the parts to be combined, such unwanted interactions can be modelled, thus having an interconnected system with predictable behaviour. Jayanthi et al. [21] experimentally tested a model system including an ATc-inducible LacI production module connected to a lac-repressible promoter with GFP downstream. This composite system was placed in a medium-copy plasmid and tested individually or in presence of a downstream “client,” including lac operator sites in a high-copy plasmid, thus providing additional binding sites for LacI. The presence of the client significantly affected the induction and deinduction dynamics. This phenomenon was captured by a mechanistic model describing the LacI-occupied DNA sites upstream of GFP and in the client binding, as a function of ATc induction.

### 2.6. Circuit Architecture

Most of the research studies described above are based on single gene cassettes. The polycistronic operon structure could be preferred when expressing genes carrying out similar functions that can be controlled by the same promoter. Although predictable RBS tuning in operons has been reported [87], the prediction of protein levels encoded by genes in operons is not trivial and cannot be simply inferred by the protein levels of individual gene cassettes. In particular, the specific operon structure can affect mRNA degradation rate and ribosome accessibility. Lim et al. developed and experimentally tested a mathematical model of transcription and translation coupling, which predicts the protein level encoded by the first gene as a function of the operon length [22]. They found and predicted protein level variations up to 2- to 3-fold. In a complementary framework, Levin-Karp et al. studied the translational coupling of an operon, that is, the mutual relationships between the translation efficiencies of neighbouring genes [102]. They individuated a >10-fold change for the protein level encoded by the second gene as a function of the translation rate of the first gene. However, the findings of Lim et al. and Levin-Karp et al. were not valid for all combinations of genes and the same phenomena were not observed in different studies [61, 102].

The measurement of mRNA levels of a transcribed operon has been useful to decouple the effects of RNA stability and translation rate change [102]. In summary, other mathematical analyses are needed to develop predictive tools that can guide biological engineers in the composition of operon structures with quantitatively predictable function, which can be inferred by the knowledge of promoter, RBSs, gene sequence, genes position, operon length, and other possible features [22].

### 2.7. Genetic Context

The context in which a gene expression cassette or a complex circuit is placed can affect its quantitative behaviour. Genetic contexts include plasmids replicating at different copy numbers per cell or the bacterial chromosome. Given a single gene expression cassette, plasmid sequence can affect promoter or terminator activity by means of the sequences flanking the cloning site, as described above for these two part classes. Moreover, intuitively, DNA copy number determines different levels of all the species (mRNA and protein), but such levels could be unpredictable, since cells may exhibit metabolic overloading when copy number is increased, thus showing nonlinear changes. This effect is commonly observed in expression cassettes at high copy number [20, 79, 103] and needs to be characterized when the cassette copy number is to be tuned. Furthermore, plasmid copy number can be intrinsically noisy [104, 105] and can also change when multiple plasmids are incorporated in the same cell [106]. To test the latter case, Lee et al. [106] showed that low copy plasmids with the heat-sensitive pSC101 replication origin maintain their copy number (about 5 copies per cell) in single plasmid systems and in 3-plasmid systems, while plasmids with the medium or high copy replication origins (p15A and ColE1, resp.) showed copy number increase when used in the 3-plasmid system compared to the single plasmid system.

Mathematical models of gene regulatory networks often use empirical Hill functions to describe activation or repression of cellular species, but DNA copy number is not explicitly present in the equations [23, 103]. For this reason, even by assuming a linear change of cellular species as a function of DNA copy number, mechanistic mathematical models should be defined to easily study the copy number effects. Although such models are also widely used to describe biochemical reactions, they are more difficult to study and identify than empirical models, thus requiring additional work to fully characterize the system of interest. Mileyko et al. used such class of models to study the copy number effects on different gene network motifs [23].

The integration of the desired expression cassette in the bacterial chromosome determines the maintenance of its DNA in a single copy, replicated with the genome. However, the quantitative behaviour of parts in the genomic context can be difficult to predict. For example, the real copy number of the desired DNA could change when integrated in different genomic positions because the sequences near the bacterial replication origin are expected to be replicated earlier than the other sequences [24, 107] and thus the specific DNA segment is actually present in the cell at a slightly higher copy number, on average. The complexity of genomic context is not limited to this effect and other not fully understood phenomena could limit the prediction of an integrated cassette. For example, transcriptional read-through from flanking genomic cassettes could affect the expression of the synthetic cassette.

## 3. Trial-and-Error Approaches

The design of a desired biological function can be achieved by randomly changing its DNA-encoded elements. In particular, promoters, RBSs, architectures, and contexts are varied, via disparate experimental methods, and the resulting circuit is screened. The success of all these methods relies on parts generation and screening efficiency, which should allow an easy and high-throughput construction and recognition of the desired phenotype [60]. Here, only representative studies are illustrated, which randomly optimize promoters, RBSs, genes, architectures, and context towards a target circuit/pathway functioning.

Promoters upstream of one or more target genes is randomly changed by directly synthesizing new promoter sequences or by assembling the genes under the control of a collection of promoters. In the first case, degenerate primers can be used to insert a new random promoter sequence upstream of a gene [108]. In the second case, promoters from existing collections of parts [55] or random fragments [109, 110] can be used in the same manner and the resulting constructs are screened. In this latter case, the characterization of promoters (or the quantification of the transcriptional activity of random fragments) is not required, because only the circuit outcome is considered to optimize the process. These two methods can be combined by producing libraries of synthetic random promoters, when required with the desired design constraints (e.g., the desired operator sites) [74, 111], that are screened by reporter genes to yield a collection of parts with diverse and graded activity; then, elements can be randomly assembled to tune the desired circuit/pathway [74, 111]. Such procedure could be partially rational: inducible promoters can be used to probe the optimal activity of a target gene and only a subset of the candidate newly generated promoters, having a constitutive activity similar to the optimal one, can be tested [20, 112, 113].

By following a similar procedure, RBSs can be randomly changed and selected. Anderson et al. [38] and Kelly [101] repaired a nonfunctional AND gate and a logic inverter, respectively, by random mutagenesis of the RBS upstream of a regulatory gene. The two gates were nonfunctional because their activity range in input did not match the activity range provided by the upstream promoter used in the final interconnected circuit. The RBS sequence mutagenesis and screening process produced circuits with the expected behaviour. The use of existing collections of RBSs can also be exploited instead of creating new ones [42, 114]. The random mutagenesis of promoters and RBSs can be performed via different widely used molecular biology methods, including error-prone PCR or DNA amplification with degenerate primers. High-throughput techniques have been recently proposed to simultaneously mutate the sequence of several elements, also in the genome, via automated procedures. The multiplex automated genome engineering (MAGE) approach was used, coupled with a microfluidic automatic system and with degenerate single-stranded DNAs to enable the lycopene pathway optimization through RBS mutagenesis for 24 target genes in plasmid or genome [115].

Genes have been randomly mutated mainly to obtain different functional protein variants with improved performance [59]. Since this approach causes amino acid variation, instead of synonymous codon replacement, the resulting protein is different. Such approaches are beyond the focus of this review. Codon change studies, without affecting protein sequence, are not widely used and they are limited to the experimental works carried out to find gene optimization rules, as described in Section 2.3 of this review. Similarly, terminators are not commonly targeted for random mutations.

When dealing with polycistronic designs, the architecture of gene expression cassettes can be randomly varied by changing the position of the genes in an operon or by flanking genes with libraries of tunable intergenic regions (TIGRs) [116]. Since the target protein level produced by genes in operons is not currently predictable, the first, intuitive, method relies on random change of gene position. This, in several studies, yielded highly diverse protein levels among the shuffled constructs. For example, bicistronic operons including the 1a-hydroxylase, adrenodoxin, and NADPH-adrenodoxin reductase genes (called ADX and ADR), used as redox partners to characterize the 25-hydroxyvitamin D3 1a-hydroxylase gene, were switched (yielding ADX-ADR and ADR-ADX constructs) and both ADR and ADX expression levels varied up to 5-fold [117]. On the other hand, the use of TIGRs relies on the assembly of various control elements (mRNA secondary structures, RNAse cleavage sites, RBS sequestering sequences, etc.) within operon genes. This random approach has proved to enable a >100-fold range of enzyme levels and a 7-fold improvement of productivity for a synthetic mevalonate pathway [116].

The genetic context can also be randomly optimized. Plasmid copy number change is an intuitive method to tune the output of circuits and pathway. Kittleson et al. [118] constructed different-allele (DIAL) strains that had the same genetic background except for an expression cassette providing different protein levels of a trans-acting replication factor (Pi or RepA); plasmids with the R6 K and ColE2 replication origins can be maintained at disparate copy number per cell levels, due to the regulation by Pi and RepA, respectively. The resulting strains were successfully used to optimize a violacein biosynthetic pathway. Considering genetic context at genomic level, different methods were used to optimize integration position and copy number of synthetic DNA-encoded production pathways via random approaches. Santos et al. developed a recombinase-assisted genome engineering (RAGE) approach, where lox sites, recognized by the Cre recombinase, are exploited to integrate very large synthetic DNA fragments into the desired genomic position, thus enabling the trial-and-error search among several predefined candidate loci [119]. They used it to optimize a 34 Kb heterologous pathway for alginate metabolism. On the other hand, the random insertion of the desired DNA parts is often carried out through transposable elements. By randomly optimizing promoter activity and genomic position at the same time, Yomano et al. optimized the expression of an ethanol production pathway [120]. In particular, they integrated a promoter-less 3-cistron ethanol production cassette in random positions of the strain of interest via a mini-Tn5 cassette (transpososome), relying on the random placement of the cassette under the control of promoters with optimal strength in the optimal genomic position.

Chromosomally integrated circuits or pathways can be also optimized by randomly changing their copy number. Methods to carry out this task rely on genomic integration of the DNA of interest together with an antibiotic resistance cassette; subsequently, recombinant strains are evolved in presence of increasing antibiotic concentration, to promote the tandem duplication of the recombinant DNA cassette, until a target efficiency is reached. This method has provided recombinant strains containing more than 25 copies of the DNA-encoded ethanol production pathway to be optimized [121, 122]. A further refinement of the methods was carried out by Tyo et al., where the chemically inducible chromosomal evolution (CIChE) was described [123]. It is analogous to the previously described procedure, but when the desired efficiency is reached the recA gene (promoting homologous recombination) is knocked out. CIChE was applied to poly-3-hydroxybutyrate (PHB) and lycopene production, yielding significant pathway improvement (4-fold and 60%, resp.). This method produced approximately 40 consecutive copies of the DNA-encoded pathway and 10-fold improvement on genetic stability [123].

## 4. Interventions on Circuit Structure to Improve Predictability

Although individual parts, networks, architectures, and contexts have the abovementioned predictability issues, several efforts have been undertaken to modify some of these elements to decrease their context-dependent variability and improve their predictability.

Davis et al. designed a set of insulated promoters that extend from −105 to +55 from the transcription start site [73]. These elements had a more predictable activity than noninsulated promoters when tested in different contexts. Mutalik et al. proposed a bicistronic design (BCD) of gene expression cassettes to effectively predict the translation initiation rate of a downstream gene [81]. This design includes a small open reading frame (ORF), with its own RBS, assembled downstream of the promoter of interest. The stop codon of this ORF is fused to the start codon of the gene of interest (thus having TAATG), which is assembled downstream. The RBS of the gene of interest is included in the small ORF upstream. With this design, inhibitory RNA structures around the gene of interest start codon or RBS are eliminated by the intrinsic helicase activity of ribosomes arriving at the stop codon of the upstream ORF. By forward-engineering an expression cassette via BCD, users should obtain the expected relative expression within 2-fold of the target value with 93% probability, which represents a great improvement over state-of-the-art predictive tools for RBSs [9, 81].

Qi et al. proposed the use of bacterial clustered regularly interspaced short palindromic repeat (CRISPR) pathway elements to engineer specific posttranscriptional cleavage of multigene operons to yield predictable expression of the individual genes, also when placed in different positions [124]. Via a complementary approach, Lou et al. used ribozymes, assembled downstream of a promoter, to improve the predictability of gene expression [125]; ribozymes cleave the mRNA eliminating their 5′ end and also act as transcription insulators.

Del Vecchio et al. [5, 126] proposed a system able to overcome retroactivity issues upon interconnection of biological systems, thus implementing a buffer (or insulator) device. It strongly relies on engineering-inspired insulators, such as noninverting operational amplifiers. The biological implementation of this mechanism includes phosphorylation-dephosphorylation reactions, which act with fast timescales, but it needs to be experimentally validated.

## 5. Conclusions

This review has described several aspects of the design of genetic circuits with predictable function. Bottom-up approaches have been recently investigated to mimic the traditional design processes in engineering areas. In this context, research studies have been carried out to evaluate the predictability boundaries of biological systems composed by precharacterized parts, providing the expected interconnection error, estimated from the study of model systems, and highlighting situations where circuits cannot behave as intended. Mathematical models support the bottom-up design steps, from the early feasibility study of complex functions to the quantitative prediction of circuit behaviour from the knowledge of basic parts function and, finally, to the debugging step.

To exploit the full potential of synthetic biology via an engineering-inspired bottom-up design of circuits, several challenges need to be faced. The main crucial issues identified in the context of this work are delineated in Box 2 in the form of outstanding questions and they are herein discussed.

Predictable biological engineering requires deepening our knowledge on context dependency and reusability of biological parts, by discovering the features that play important roles in parts function predictability. Technology advances in the DNA synthesis field can support the testing of large number of hypotheses by providing huge libraries of constructs at affordable price. In fact, although large-scale studies have been reported to support the investigation of different aspects of parts predictability [60, 81, 96], the cost and scale of DNA synthesis are still a major bottleneck for basic research, since many studies require a very large number of construct variants, as in the case of codon usage dependency in protein expression [88]. The development of high-throughput methods for parts measurement plays a complementary role, because multifaceted characterization of parts performance needs to be carried out. In particular, to fully characterize the activity of parts, the simultaneous quantification of DNA, RNA, and proteins is required to accurately decouple effects due to circuit copy number, transcription, and translation, to improve the knowledge of all the atomic steps involved in parts function. In addition, ad hoc experimental designs, data analysis tools, and mathematical models can support the above procedures; for example, models can be of help in the estimation of nonobservable parameters, useful to characterize parts function [36].

Empirical mathematical models of gene regulatory networks are currently used to summarize the function of parts and predict the quantitative behaviour of higher-order devices. Although they are widely used, in some cases mechanistic models could be more appropriate tools, such as in the study of DNA copy number variations or retroactivity effects. Other tools enable the prediction of parts activity from the knowledge of their nucleotide sequence. Although promising results have been obtained, particularly in the case of RBSs that are already optimized via these computational methods, these tools need to be significantly improved. The data and knowledge gained in the above “discovery” step are to be exploited in the development of predictive computational tools with greater accuracy than the current ones. In this context, novel tools can be based on the acquired biological knowledge, which will be used to define essential rules for parts function prediction or can be data-based, where machine learning methods are used to learn the relationships of interest for parts prediction. Context-dependent activity change of individual parts and mathematical models of interconnected networks should ultimately be integrated to contribute unique tools for interconnected circuit design from parts sequence.

In addition to existing parts prediction, an ambitious goal of synthetic biology is the construction of unnatural parts with finely tuned customized function. To this aim, the computational design tools need to be expanded to support the forward engineering of new components according to specific design rules, learned from data examples or from the acquired biological knowledge. Again, the currently available RBS design tools already enable the design of RBSs with desired strength, given the downstream gene sequence, although their performance needs to be significantly improved [57]. Specifically, the RBS Calculator computes novel RBS sequences with about 47% chance to show the target strength within 2-fold [81].

Even though most of our current biological knowledge is based on population-averaged data and central tendency values, cell-to-cell variability is a crucial issue and can bring to unpredictable system behaviour. Although the main aspects of this point are described elsewhere [127] and are beyond the scope of this review, we want to highlight that biological noise can be detrimental for circuits function, even when central tendency values are predictable. For this reason, the full characterization of biological components should also take into account cell-to-cell variability, which needs to be propagated throughout an interconnected network of well-characterized modules to obtain reliable quantitative predictions of network output.

In this review, trial-and-error approaches involving the random-based optimization of parts/circuit function have also been briefly illustrated. These approaches rely on affordable parts construction methods and efficient high-throughput-compatible screening methods to select the best combination of genetic parts, while these approaches cannot be efficiently applied when this condition does not persist. The technology advances mentioned above could greatly support the generation of large libraries to be screened via appropriate high-throughput measurement techniques, even without significant improvements in biological discoveries about context-dependent variability. However, while the learning of predictability boundaries is expected to contribute definitive predictive tools to handle the complexity of biological systems, trial-and-error approaches do not ensure the success of synthetic biology. In fact, large numbers of candidate constructs can be built up, but high-throughput measurement methods are not always available for the quantitative evaluation of circuit activity and the impact of pure trial-and-error approaches remains limited to specific projects. For this reason, bottom-up approaches urgently need to be refined to exploit the full potential of synthetic biology. A mixture of prediction tools, even with nonoptimal accuracy, and trial-and-error approaches could rapidly boost the efficiency of biological engineering, by providing a smaller search space than fully random-based approaches.

Finally, intense interventions on genetic circuits have been reported, which can provide considerable improvements to the predictability of promoters, RBSs, architecture, and retroactivity issues in different contexts. Since such improvements are highly promising, these modifications should be used in different studies to demonstrate their benefits on large scale and they should be considered in all the previously mentioned issues.

## Figures and Tables

**Box 1:**
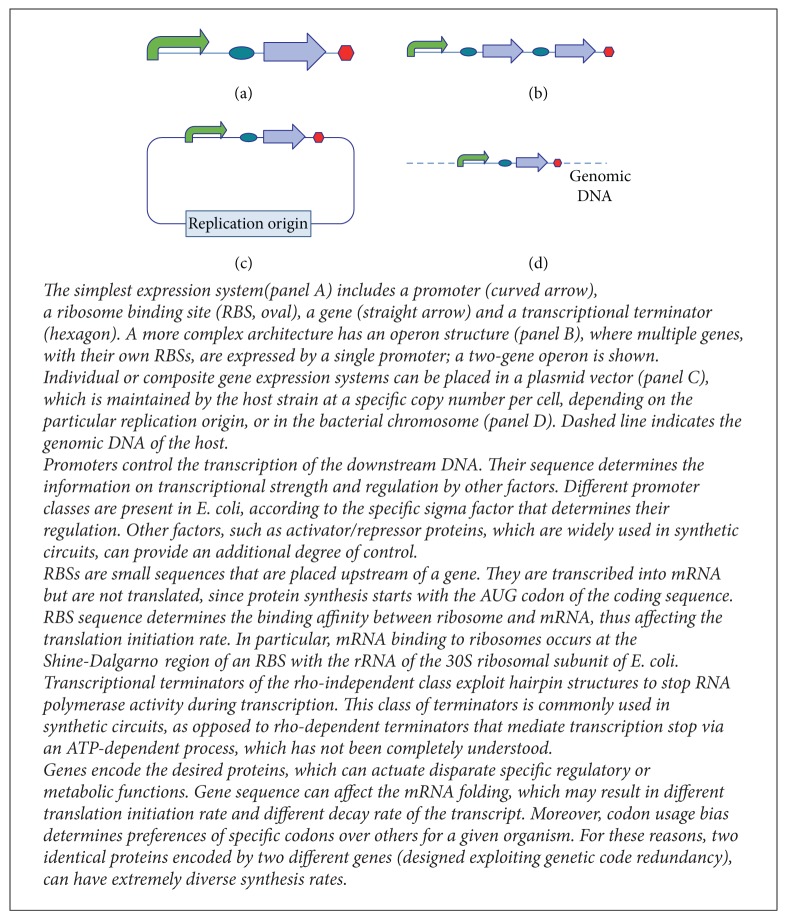
Genetic parts and architecture of a gene expression system.

**Box 2:**
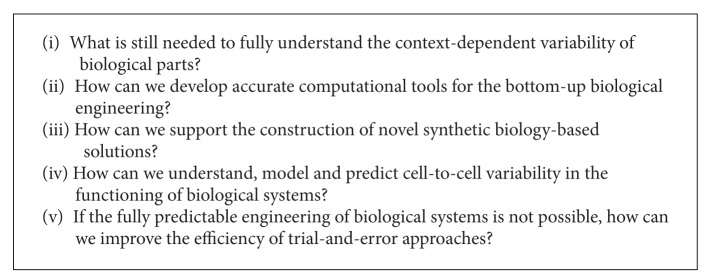
Outstanding questions.

**Table 1:** Selected computational methods and tools that support the bottom-up design in biological engineering.

Part, architecture or context	Description	Reference
Promoters	Strength prediction tool for sigmaE promoters, using a position weight matrix-based core promoter model and the length and frequency of A- and T-tracts of UP elements.	[6]
	Strength prediction tool for sigma70 promoters, using partial least squares regression.	[7]

Promoter-RBS pairs	Strength prediction tool for sigma70 promoter-RBS pairs, using an artificial neural network.	[8]

RBSs	RBS Calculator: a web-based tool for RBS strength prediction and forward engineering, frequently updated and able to design RBS libraries.	[9]
	RBS Designer: a stand-alone tool for RBS strength prediction and forward engineering, it considers long-range interactions within RNA and it can predict the translation efficiency of mRNAs that may potentially fold into more than one structure.	[10]
	UTR Designer: a web-based tool for RBS strength prediction and forward engineering, able to design RBS libraries and with the codon editing option to change RNA secondary structures.	[11]

Genes	GeMS: web-based tool for gene design, using a codon optimization strategy based on codon randomization via frequency tables.	[12]
	Optimizer: web-based tool for gene design using three possible codon optimization strategies: “one amino acid-one codon”, randomization (called “guided random”) and a hybrid method (called “customized one amino acid-one codon”).	[13]
	Synthetic Gene Designer: web-based tool for gene design with expanded range of codon optimization methods: full (“one amino acid-one codon”), selective (rare codon replacement) and probabilistic (randomization-based) optimization.	[14]
	Gene Designer: stand-alone tool for gene design using a codon randomization method based on frequency tables and with the possibility to filter out secondary structures and Shine-Dalgarno internal motifs.	[15]

Terminators	Termination efficiency prediction tool based on a linear regression model using a set of sequence-specific features identified via stepwise regression.	[16]
	Termination efficiency prediction tool based on a biophysical model using a set of free energies, previously identified as important features.	[17]

Interconnected networks	A range of empirical or mechanistic ODE or steady-state models can be used to predict complex systems behaviour from the knowledge of individual parts/devices parameters.	[5, 18–21]

Architecture	Protein expression prediction for the first gene of an operon, given the downstream mRNA length, via a linear regression model.	[22]

Context	Mechanistic ODE models where the DNA copy number is explicitly represented.	[23]
	Protein expression prediction tool, based on linear regression model, given the chromosomal position of the gene and its orientation.	[24]
